# Advances in Osteoblast and Mitochondrial Dynamics and Their Transfer in Osteoporosis

**DOI:** 10.1111/jcmm.70299

**Published:** 2024-12-19

**Authors:** Jiaxuan Liu, Xuequan Zhang, Honghao Hou, Jun Ouyang, Jingxing Dai

**Affiliations:** ^1^ Guangdong Provincial Key Laboratory of Digital Medicine and Biomechanics & Guangdong Engineering Research Center for Translation of Medical 3D Printing Application & National Virtual & Reality Experimental Education Center for Medical Morphology (Southern Medical University) & National key Discipline of Human Anatomy, School of Basic Medical Sciences Southern Medical University Guangzhou China; ^2^ Heyuan Hospital of TCM Heyuan China

**Keywords:** mitochondrial dynamics, mitochondrial transfer, osteoblasts, osteoclasts, osteoporosis

## Abstract

Mitochondria are important organelles in the human body and play a major role in providing cellular energy, maintaining tissue homeostasis and apoptosis. Osteoporosis, characterised by a decrease in the amount of bone tissue per unit volume, is a metabolic bone pathology with multiple causes. Under pathological conditions, mitochondrial dysfunction leads to an imbalance in mitochondrial homeostasis, resulting in a disruption of osteoblast–osteoclast homeostasis, which in turn disrupts bone homeostasis, and this disruption of homeostasis is an important pathogenetic mechanism underlying chronic metabolic bone disease in osteoporosis. Numerous studies have shown that bone homeostasis is closely related to mitochondrial dynamics and mitochondrial translocation in the mitochondrial quality control system, and the balance between osteoblasts and osteoclasts is closely related to osteoporosis. In this review, we describe the progress of osteoblast and osteoclast research and mitochondrial dynamics in osteoporosis, and the role of mitochondrial translocation in bone homeostasis, in the hope that it can stimulate new research in osteoporotic metabolic bone disease and the development of novel therapeutic strategies.

**Abbreviations**
AMPK: AMP‐activated protein kinase
Cd: cadmium
DNM1L: dynamin 1‐like protein
DRP1: Drp1‐dynamin‐related protein 1
EV: extracellular vesicle
FIS1: mitochondrial fission 1
IL‐6: Interleukin‐6
MDVs: mitochondria‐derived vesicles
MFF: mitochondrial fission factor
MFN1: mitochondrial fusion protein 1
MFN2: mitochondrial fusion protein
MSCs: mesenchymal stem cells
mtDNA: mitochondrial DNA
NF‐κB: nuclear factor‐κB
OP: osteoporosis
OPA1: optic nerve atrophy protein 1
PGC1α: proliferator‐activated receptor‐γ coactivator 1 α
RANKL: NF‐κB ligand receptor activator
ROS: reactive oxygen species
SASP: senescence‐associated secretory pathway
SIRT1: senescence‐resistant enzyme 1
TNF‐α: tumour necrosis factor α
TNT: tunnelling nanotubes
VSMC: vascular smooth muscle cells

## 1 Introduction

Osteoporosis (OP) defines a group of bone diseases with multiple causes, with normal calcification of bone tissue and a normal ratio of calcium salts to matrix. It is a metabolic bone disease that is characterised by a decrease in the amount of bone tissue per unit of volume [1]. In most cases of osteoporosis, the decrease in bone tissue is mainly due to increased bone resorption. In recent years, osteoporosis is no longer a disease unique to older patients, and may involve nutritional osteoporosis caused by protein, vitamin C or calcium deficiency. The weakening of mechanical stimulation of the skeleton can be caused by muscle atrophy, bone formation, bone resorption and increased osteoporosis, as well as the unknown causes of most young people in youth‐type osteoporosis [1, 2]. Therefore, the reduction of the incidence of OP and the elucidation of its pathogenesis have become urgent concerns.

Mitochondria, a two‐membrane‐covered organelle present in most eukaryotic cells, is an energy‐producing structure in the cell and is the primary site of aerobic respiration [3]. Mitochondria possess their own genetic material and inheritance system, but their genome is limited in size, and thus are considered semiautonomous organelles. In the physiological state, mitochondria can not only provide energy to the cell, but they also participate in cell differentiation, cellular information transfer, apoptosis, and other processes, and can regulate cell growth and the cell cycle [3]. When mitochondrial dysfunction occurs, it leads to intracellular and extracellular disorders, disrupting the balance between osteoblasts and osteoclasts, which can lead to the development of osteoporosis [4, 5, 6, 7]. This paper reviews mitochondrial dynamics in osteoblasts and osteoclasts and mitochondrial dysfunction in osteoporosis from the point of view of mitochondrial dynamics and the role of mitochondrial transfer in the homeostasis of bone.

## 2 Core Molecular Mechanisms of Mitochondrial Dynamics

Mitochondrial dynamics is a unique system in which the control of mitochondrial shape and size are two important influences on mitochondrial activity. Mitochondria respond to the requirements of the cellular environment by constantly dividing and merging to change their morphology and maintain function [8]. Drp1‐Dynamin‐related protein 1 (DRP1) encodes a dynamin‐like GTPase that regulates mitochondrial dynamics and peroxisomal fission by translocating to the outer mitochondrial membrane and undergoing a process of oligomerisation. DRP1 mediates membrane fission through the initiation of oligomerization and the formation of membrane‐associated tubular structures. These tubular structures wrap around the site of the fracture and contract and sever the mitochondrial membrane by a mechanism that depends on the hydrolysis of GTP [9]. Optic nerve atrophy protein 1 (OPA1) is one of the factors that control mitochondrial fusion, maintenance of mitochondrial DNA (mtDNA), bioenergetics and cristae integrity [10]. OPA1, also known as mitochondrial dynamin‐like protein, is a 120 kDa protein divided into two active forms, long OPA1 and short OPA1. These OPA1 proteins are anchored to the inner mitochondrial membrane by protease hydrolysis under physiological conditions, and together they are involved in the fusion of the inner mitochondrial membrane [11]. Mitochondrial fission is the separation of damaged mitochondrial structures from intact mitochondria, whereas mitochondrial fusion is the connection and mixing of neighbouring depolarised mitochondria.

**FIGURE 1:**
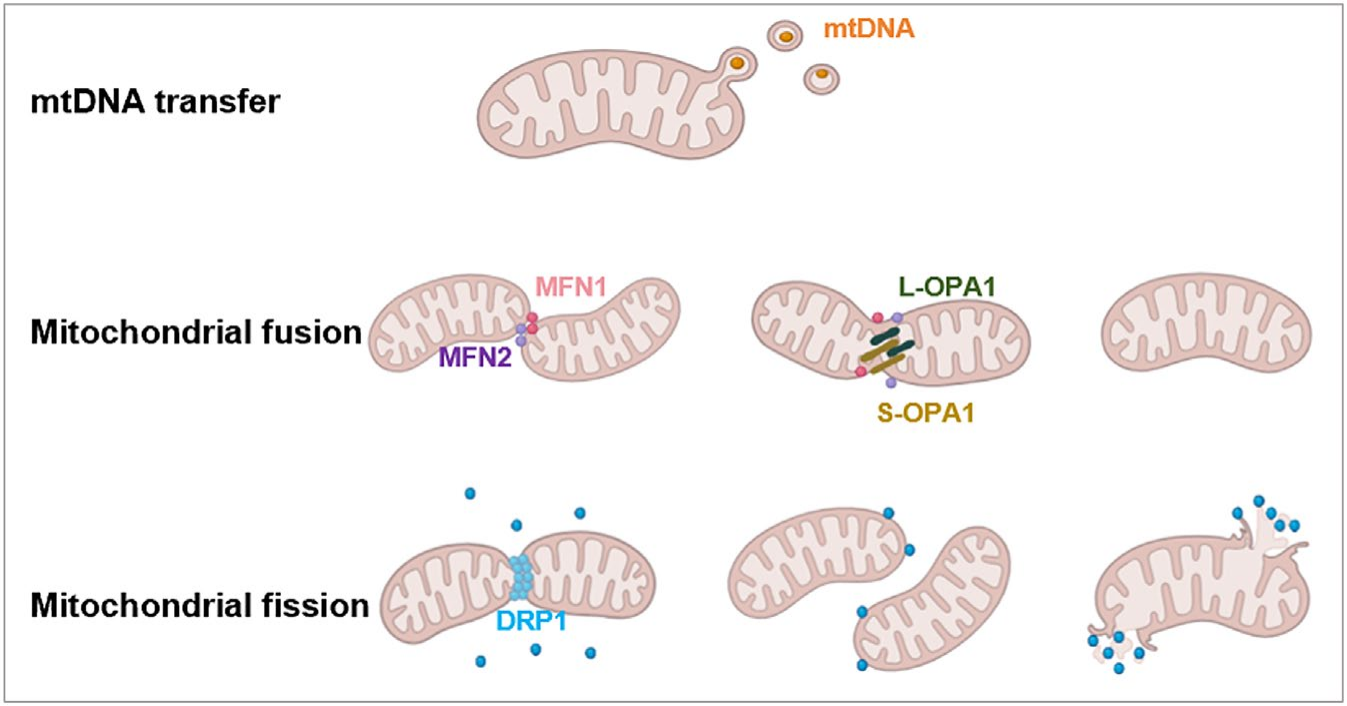
Mitochondrial outer membrane fusion is mediated by Mfn1/2, whereas inner membrane fusion requires OPA1 participation. The long L‐OPA1 and short S‐OPA1 of mitochondrial dynamoprotein‐like proteins participate in mitochondrial inner membrane fusion. The DRP1‐mediated mitochondrial fission process is realised through translocation and oligomerisation of the mitochondrial outer membrane. Mitochondria are secreted to recipient cells by extracellular vesicles (EVs). They are internalised by target cells through receptor‐mediated endocytosis, direct diffusion or other mechanisms. Extracellular vesicles can carry the complete mitochondrial genome. These EVs, in turn, can transfer their mtDNA into metabolically damaged cells, thereby restoring metabolic activity.

Changes in mitochondrial morphology are regulated by fusion protein (mitochondrial fusion protein 1, MFN1; mitochondrial fusion protein 2, MFN2) [12] and fission proteins (dynamin 1‐like protein, DNM1L; mitochondrial split protein 1 protein, FIS1). These factors regulate the turnover of the mitochondria by facilitating the dilution and removal of the damaged organelles. MFN1 or MFN2 are located on the outer membrane of the mitochondria and mediate the fusion of the mitochondrial outer membrane, while OPA1 is involved in mitochondrial inner membrane fusion and maintenance of mitochondrial cristae structure and integrity [13]. Mitochondrial morphology and respiration participate in mitochondrial biogenesis, and in osteogenic differentiation and are promoted in mouse osteoblast precursors in the MFN2 knockout mouse mode [14]. The major protein that regulates mitochondrial fission is DRP1, which is mainly found in the cytoplasm of cells, and is recruited to mitochondria and oligomerized by binding mitochondrial fission factor (MFF) receptors, Fis1, 49 kDa mitochondrial dynamin (MiD49), and 51 kDa mitochondrial dynamin (MiD51), resulting in hydrolysis of GTP through the GTPase and disruption of the inner and outer mitochondrial membranes [15]. Therefore, DRP1 plays a key role in mitochondrial dynamics; DRP1 induces excessive mitochondrial fission [16]. When the homeostasis of fission and fusion is disrupted, the mitochondrial quality control system is disrupted, resulting in mitochondrial dysfunction and cell death, which ultimately leads to tissue damage and diseases such as osteoporosis and intervertebral disc degeneration [17] (Figure 1).

### 2.1 Osteoblast and Mitochondrial Dynamics in Osteoporosis

When mitochondrial homeostasis is disrupted by fissioning and fusing, the balance of osteoblasts and osteoclasts is disrupted, and the development of osteoporosis is accelerated. Minor perturbations lead to homeostatic adaptation, whereas major perturbations can cause damage to the cell, leading to apoptosis or necrosis and death [4]. In most cells, mitochondria, endoplasmic reticulum and peroxisomes are the main sources of reactive oxygen species (ROS). Dysfunctional mitochondria produce large amounts of ROS, which damage nuclear DNA and activate the 5′‐adenylate activated protein kinase (AMPK) signal. Activation of AMPK pathway inhibited the activity of NAD‐dependent protein deacetylase anti‐ageing enzyme 1(SIRT1). Reduced levels of NAD^+^ under oxidative stress conditions further influence SIRT1, leading to reduced levels of proliferator‐activated receptor‐γ coactivator 1 α (PGC1α), which ultimately reduces mitochondrial biosynthesis [18]. Some studies had indicated that knockdown of SIRT1 results in reduced bone mineral density and an imbalance in osteoblasts/osteoclasts ratio [19, 20]. This is indirect evidence for the imbalance of osteoblast/osteoclast ratio caused by disruption of mitochondrial homeostasis. Ding et al. [5], demonstrated that disruption of mitochondrial homeostasis directly affects intercellular communication by demonstrating that impaired mitochondrial transfer from bone line cells alters the protection of glutathione metabolism and promotes osteoclast activity.

Under physiological conditions, ROS can be involved in the regulation of normal body functions such as cell adhesion, programmed cell death, and signal transduction of membrane receptors. However, ROS can also damage all major cellular components, such as mitochondria and their inner and outer membranes, at high concentrations. The antioxidant defence system can prevent the balance from being disturbed, thus preventing oxidative stress. Under oxidative stress, DRP1 and its phosphorylation state increased in osteoblasts, and mitochondrial fragmentation, aberration and vesicular changes occurred in osteoblasts [15]. Smirnova et al. [21] demonstrated that over‐up‐regulation of Drp1 and its abnormal distribution eventually led to abnormal mitochondrial fission, which is harmful to cells. DRP1 also plays a key role in ROS‐induced cell dysfunction. Gan et al. [22, 23] used pharmacological inhibitors of Drp1 to restore mitochondrial function and improve cell damage caused by ROS. Gan et al. [15] also demonstrate that blockade of Drp1 attenuates oxidative stress‐induced osteoblast dysfunction. However, irisin has been shown to inhibit mitochondrial dysfunction due to mitochondrial fission by activating AMPK and decreasing Drp1 expression and translocation to mitochondria, as well as by inhibiting the transformation of osteoblasts from VSMC [24]. Zhang et al. [16] demonstrated that tumour necrosis factor (TNF‐α) promoted excessive accumulation of ROS in mitochondria, while up‐regulating DRP1 caused the mitochondrial membrane potential to collapse, which in turn led to mitochondrial vesiculation and fragmentation, reduced mitochondrial function and inhibiting osteoblast activity. Glucose reduced the migratory potential and chemotaxis of osteoblasts and decreases associated cell signalling.

High glucose levels also led to a shift in mitochondrial dynamics towards more fused and less fragmented mitochondria, along with a reduced expression of DRP1, suggesting a reduction in mitochondrial biogenesis [25]. Thus, oxidative stress induces osteoblast dysfunction through DRP1‐mediated mitochondrial hyper‐fragmentation, but irisin improves this effect by reducing Drp1 expression. Several studies have directly demonstrated that exposure to the physical factor cadmium (Cd) is also a possible cause of osteoporosis, and Cd has direct toxic effects on osteoblasts, osteoclasts, and bone metabolism, among which osteoblasts are the main targets of Cd toxicity. Cd inhibits Nrf2/NQO1 via activation of the PERK‐EIF2α‐ATF4‐CHOP signalling pathway, leading to changes in the morphology and structure of MC3T3‐E1 cells, loss of original mitochondrial shape, mitochondrial swelling, significant vacuolation and crest breakage, thereby achieving osteogenic inhibition and contributing to the development of osteoporosis. However, N‐acetyl‐L‐cysteine and 4‐phenylbutyric acid can effectively block Cd‐induced damage to MC3T3‐E1 [26]. This provides new ideas and methods for the treatment of Cd‐related bone diseases.

### 2.2 Osteoclast and Mitochondrial Dynamics in Osteoporosis

Mitochondrial dysfunction not only affects osteoblast function but also has an impact on osteoclast differentiation. When osteoclast activity exceeds that of osteoblasts, it leads to osteoporosis with reduced bone microstructure, decreased bone mass, and increased incidence of fractures.

AMPK‐activated protein kinase α1 (AMPKα1) is a highly expressed energy sensor in osteoclasts and is involved in metabolic remodelling during osteoclast differentiation and activation. AMPKα1 regulates mitochondrial fusion and fission markers, up‐regulating Mfn2 and down‐regulating DRP1. Ribeiro et al. [27] have shown that AMPKα1 in osteoclasts has a protective effect on the integrity of bone microstructure. Osteoclasts lacking AMPKα1 showed increased expression of mitofusin 1 (MFN1) and mitofusin 2 (MFN2), two major genes involved in mitochondrial fusion. AMPKα1‐deficient osteoclasts showed an increase in the osteoclast marker (NFATc1 and DC‐STAMP) and the fusion marker Mfn2 (Table 1). In addition, mitochondrial binding is the main MFN2 mechanism of osteoclast formation in vitro. Mitochondrial binding is the main MFN2 mechanism of osteoclast formation in vitro. Ballard A et al. [28] demonstrated that MFN2‐mediated restoration of ER‐mitochondrial contact modulates mitochondrial Ca^2+^ uptake to produce large fluctuations in cytoplasmic Ca^2+^, thereby promoting osteoclastogenesis. Osteoclast generation was inhibited in MFN1/2‐deficient cultures. In mitochondrial dynamics, in addition to the influence of fusion molecules on osteoclast differentiation and activity, mitochondrial mitogen‐like protein DRP1 can down‐regulate the key transcription factors of osteoclast formation, c‐Fos and NFATc1, to inhibit osteoclast differentiation by knocking down or using DRP1 inhibitor Mdivi1. Jeong et al. [29], used DRP1 inhibitors in a cranial model and showed that the process inhibited lipopolysaccharids‐induced osteoclast formation and reduce the bone loss induced by oophorectomy.

**TABLE 1:** Dynamic relationship between osteoclasts and mitochondria.

Molecular	Mitochondrial dynamic pathway	Molecular pathway	Cellular influence in osteoporosis
AMPKα1↑	MFN2↑, DRP1↓	—	Osteoclast activity↓ [27]
—	MFN1, MFN2↓	Ca^2+^–NFATc1	Osteoclast↓ [28]
—	DRP1↓	c‐Fos‐NFATc1	Osteoclast↓ [29]

## 3 Mitochondrial Transfer

Mitochondrial transfer is an important mechanism regulating cell‐to‐cell interactions and adaptation to the outside world and can be broadly categorised under a fourth aspect of mitochondrial dynamics, subcellular organelle transport.

(1) Mitochondrial transport along microtubules: In 2004, Rustom et al. [30] found that mitochondrial transport is possible through tunnelling nanotubes (TNT), whereby mitochondria are distributed in the same direction as microtubules and migrate in the cytoplasm to areas of high functionality using microtubules as a trajectory powered by motor proteins. Mitochondria are transported along microtubules by connecting them to motors and motor proteins via Miro1 and Miro2 RHO‐GTPase. Some key cellular functions, such as cell proliferation, have been shown to be regulated by Miro proteins fine‐tuning actin and microtubule‐dependent mitochondrial movement and localisation [31].

(2) Mitochondria are delivered to receptor cells by secretion of EVs, which are internalised by target cells through receptor‐mediated endocytosis, direct diffusion, or other mechanisms. EVs can carry the complete mitochondrial genome. These EVs can, in turn, transfer their mtDNA to metabolically impaired cells, thus restoring metabolic activity. Suh et al. [32] demonstrated that mature osteoblasts promote bone progenitor cell differentiation by secreting fragmented mitochondria and mitochondrial‐derived vesicles (MDVs) formed from mitochondrial donut forms. In contrast, Morrison et al. [33] describes the promotion of anti‐inflammatory activities by mesenchymal stem cells (MSCs) through EV‐mediated mitochondrial transfer in the context of acute respiratory distress syndrome.

(3) Mitochondrial transfer by mitochondrial extrusion. Nakajima et al. [34] described mitochondrial extrusion for the first time. In this study, mitochondrial extrusion is a process in which cytoplasmic vacuoles originating in the plasma membrane engulf broken mitochondria and squeeze them into the extracellular space, which requires a complete cellular scaffold consisting of actin and microtubules.

(4) Mitochondrial transfer by cell fusion. This modality has been less studied in osteoblast‐related studies.

Mitochondrial transfer is essential for the maintenance of bone homeostasis. Specialised osteoblasts, called osteocytes embedded in mineralised bone, transfer mitochondria to each other through a network of interconnected dendrites [35]. Osteoblasts and bone progenitor cells connect through mitochondria in dialogue with each other to meet metabolic demands, which can optimise bone mineral homeostasis and health. Mitochondria are secreted from mature osteoblasts to promote differentiation of bone progenitor cells [32]. The construction of an osteogenic induction group with OPA1 knockdown or enhanced overexpression of Fis1 can stimulate mitochondrial fragmentation, donut formation, and mitochondrial secretion through the CD38/cADPR signalling pathway and can accelerate osteogenesis [32]. Furthermore, this paper confirmed that mitochondrial fusion promoter M1 induces OPA1 expression and inhibits osteogenesis, while osteoblast‐specific OPA1 deletion has been shown to increase bone mass [32]. Osteocytes inhibit cancer bone metastasis by transferring mitochondria to cancer cells, which induces cGAS/STING‐mediated anti‐tumour immunity. MIRO1 and MFN2 mediate intercellular mitochondrial transfer, and the efficiency of this transfer decreases with age (and MFN2 levels). Osteoblast‐specific deletion of Rho GTPase 1 or MFN2 inhibits intercellular mitochondrial transfer, reduces immune cell infiltration and promotes bone metastasis growth [36].

**FIGURE 2:**
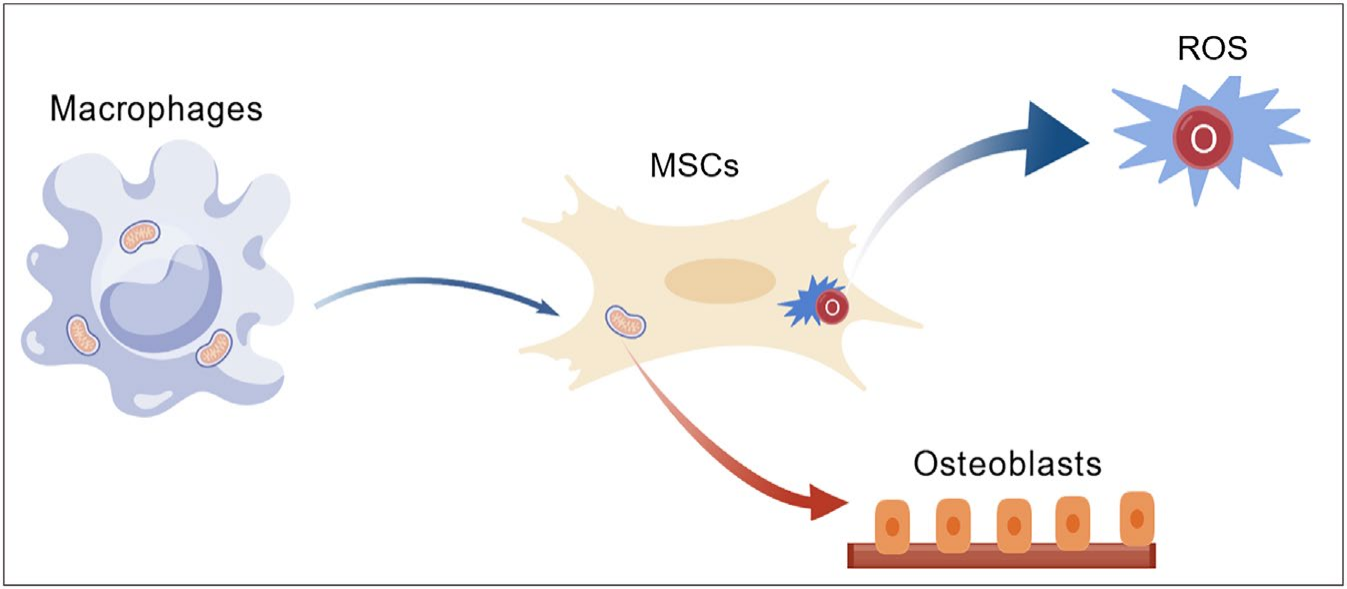
Macrophages in the bone marrow environment deliver mitochondria to the MSCs to promote osteogenic differentiation of the MSCs, while the fluid triggers a burst of reactive oxygen species in the MSCs, leading to metabolic remodelling of the MSCs.

**FIGURE 3:**
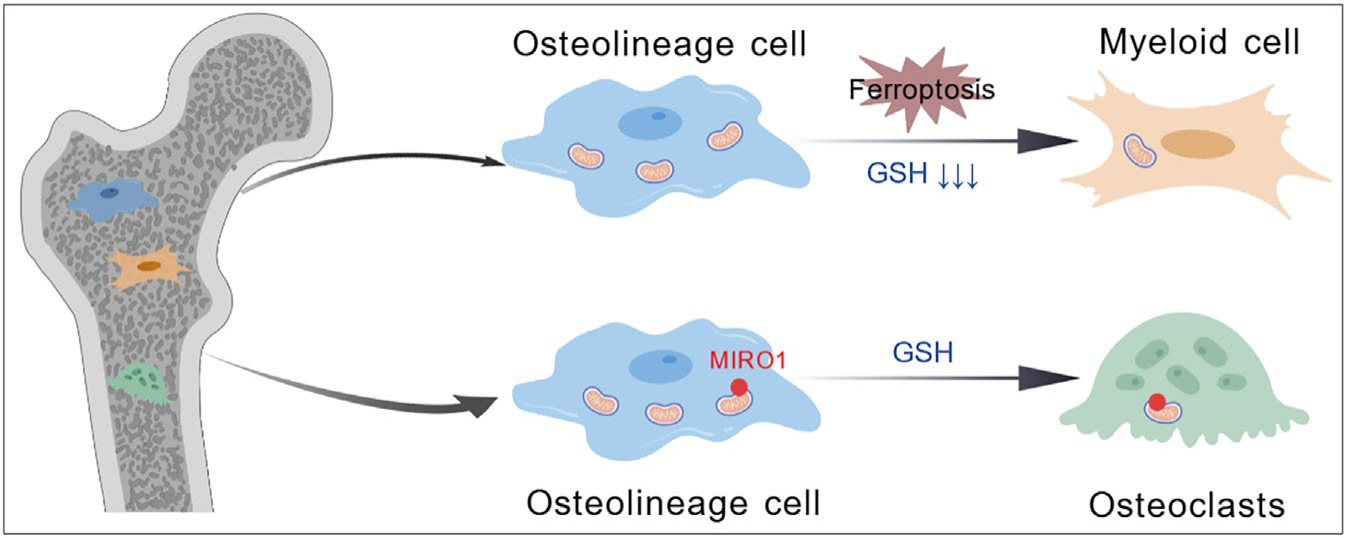
Mitochondria from bone line cells regulate bone marrow cell‐mediated bone resorption by altering iron death regulated by glutathione (GSH) metabolism, primarily by reducing the progression of GIOP through depletion of GSH. At the same time, when bone line cells transfer mitochondria to osteoclasts, they also inhibit cell activity and change GSH metabolism in osteoclasts, and this process is mediated by MIRO1.

In osteoporosis, mitochondrial transfer between different cells can regulate bone homeostasis through metabolic crosstalk. Cai et al. [37] found that macrophages in the bone marrow environment promote the osteogenic differentiation of MSCs by delivering mitochondria to MSCs, and the metabolic remodelling of MSCs in the bone marrow is precisely the mitochondria transferred by M1‐type macrophages that are received and then trigger the outbreak of reactive oxygen species (Figure 2). Ding et al. [5] found that for glucocorticoid‐induced osteoporosis (GIOP), mitochondria from bone line cells regulate bone marrow cell‐mediated bone resorption by altering iron death regulated by glutathione (GSH) metabolism, and mitochondrial transfer from bone line cells to bone marrow cells also contributes to GSH depletion. This in turn mitigated GIOP's progress. Ding et al. [5] further proved that when bone line cells transfer mitochondria to osteoclasts, they inhibit their cell activity and change GSH metabolism in osteoclasts, and MIRO1 mediates the transfer process of mitochondria from bone line cells to osteoclasts (Figure 3).

## 4 Conclusions and Outlook

This article reviews the role of mitochondrial dynamics in the development and progression of osteoporosis, with emphasis on the mechanisms of key molecules and signalling pathways in mitochondrial dynamics that affect bone homeostasis and bone metabolism. The regulatory mechanism of molecules related to mitochondrial dynamics plays an important role in the genesis and development of osteoblasts, osteoclasts, and osteoporosis. At present, mitochondrial transfer in osteoporosis diseases involves mitochondrial transfer from macrophages to mesenchymal stem cells, mitochondrial transfer from osteoblasts to osteoclasts, mitochondrial transfer from other osteoblasts to osteoclasts, and specific transfer modes. Whether other types of intercellular communication exist in osteoporosis remains unclear. Further research on the specific pathways of mitochondrial transfer between cells, or whether there are other organelles transfer, will provide new ideas for future research.

## Author Contributions

**Jiaxuan Liu:** conceptualization (equal), writing – original draft (equal), writing – review and editing (equal). **Xuequan Zhang:** conceptualization (equal), writing – original draft (equal), writing – review and editing (equal). **Honghao Hou:** conceptualization (equal), writing – review and editing (equal). **Jun Ouyang:** conceptualization (equal), writing – review and editing (equal). **Jingxing Dai:** conceptualization (equal), funding acquisition (lead), project administration (lead), supervision (lead), writing – review and editing (lead).

## Ethics Statement

The authors have nothing to report.

## Conflicts of Interest

The authors declare no conflicts of interest.

## Data Availability

The authors have nothing to report.
